# Divergent biology and outcomes of somatic transformations in germ cell tumors

**DOI:** 10.1093/oncolo/oyag253

**Published:** 2026-06-30

**Authors:** Zachariah Thomas, Andrew C Johns, Michael Glover, Emanuele Crupi, Yago L Nieto, John F Ward, Jose A Karam, Wayne L Hofstetter, Monica Desai, Seungtaek L Choi, Amishi Y Shah, John Lin, Mohammad Jad Moussa, Cindy Y Jiang, Omar Alhalabi, Matthew T Campbell

**Affiliations:** Department of Genitourinary Medical Oncology, The University of Texas MD Anderson Cancer Center, Houston, TX 77030, United States; Department of Genitourinary Medical Oncology, The University of Texas MD Anderson Cancer Center, Houston, TX 77030, United States; Department of Genitourinary Medical Oncology, The University of Texas MD Anderson Cancer Center, Houston, TX 77030, United States; Department of Genitourinary Medical Oncology, The University of Texas MD Anderson Cancer Center, Houston, TX 77030, United States; Department of Stem Cell Transplantation and Cellular Therapy, The University of Texas MD Anderson Cancer Center, Houston, TX 77030, United States; Department of Urology, The University of Texas MD Anderson Cancer Center, Houston, TX 77030, United States; Department of Urology, The University of Texas MD Anderson Cancer Center, Houston, TX 77030, United States; Department of Thoracic and Cardiovascular Surgery, The University of Texas MD Anderson Cancer Center, Houston, TX 77030, United States; Department of Genitourinary Medical Oncology, The University of Texas MD Anderson Cancer Center, Houston, TX 77030, United States; Department of GU Radiation Oncology, The University of Texas MD Anderson Cancer Center, Houston, TX 77030, United States; Department of Genitourinary Medical Oncology, The University of Texas MD Anderson Cancer Center, Houston, TX 77030, United States; Department of Genitourinary Medical Oncology, The University of Texas MD Anderson Cancer Center, Houston, TX 77030, United States; Internal Medicine Residency Program, Baylor College of Medicine, Houston, TX 77030, United States; Department of Genitourinary Medical Oncology, The University of Texas MD Anderson Cancer Center, Houston, TX 77030, United States; Department of Genitourinary Medical Oncology, The University of Texas MD Anderson Cancer Center, Houston, TX 77030, United States; Department of Genitourinary Medical Oncology, The University of Texas MD Anderson Cancer Center, Houston, TX 77030, United States

**Keywords:** germ cell tumor, somatic-type malignancy, teratoma, malignant transformation

## Abstract

**Purpose:**

Somatic-type malignancy (SM) of germ cell tumor (GCT) is rare but demonstrates aggressive behavior. Differences by primary site, time to transformation, and mutational status remain poorly characterized.

**Patients and Methods:**

We reviewed all SM patients between June 2016 and May 2025 at a tertiary referral center. Time to somatic transformation (TST) was defined from the initial GCT diagnosis to SM detection. SM were classified based on time of detection- at initial diagnosis (*de novo*), at consolidative surgery, at relapse within 5 years, and evolved SM at relapse after 5 years. Descriptive statistics, Kaplan-Meier estimates, and Cox regression analyses were used.

**Results:**

72 patients were identified: 40 (56%) with testicular, 24 (33%) with mediastinal, and 8 (11%) with other primaries. 22/24 (92%) mediastinal tumors with SM had sarcomatous transformation. Sarcoma was seen in 25/42 (60%) of *de novo* SM while adenocarcinoma was detected as an *evolved* entity in 5/6 (83.3%) cases. Embryonic type neuroectodermal tumor (ENET) histology carried poor prognosis compared to non-ENET histology (median, OS 1.8 vs 8 years; HR, 1.94; *P* = 0.1). Genomic data available showed *PTEN-AKT-mTOR* pathway mutations (33%) and *TP53* mutations (33%), enriched in extra-gonadal sarcomatous SM.

**Conclusions:**

SM exhibits temporal, histologic, and molecular distinctions across primary sites. Response to frontline therapy and survival outcomes are poor. Future direction includes the exploration of underlying predictors of transformation and identification of targetable alterations in the relapsed setting.

Implications for Practice This report highlights distinct histological, molecular, and evolutionary patterns among somatic-type malignancies (SM) of germ cell tumors. We report an enrichment of sarcomatous dedifferentiation among mediastinal SM that is detected upfront. In contrast, SM-type adenocarcinoma is an evolved entity, emerging decades after initial GCT diagnosis. Extragonadal SM is enriched for *TP53* mutations. Atypical and midline presentation of somatic malignancies should raise suspicion for a transformed germ cell tumor.

## Introduction

Somatic-type malignancy (SM) arising within a germ cell tumor (GCT) is a rare but highly aggressive entity, occurring in approximately 3% of all GCTs.^1^ Initially described by Ulbright et al in 1984, SM is characterized by overgrowth and infiltration of somatic components within a teratoma, displacement of adjacent elements, loss of typical GCT cell surface markers, and lack of production of traditional serum tumor markers including beta-human chorionic gonadotropin (*β-hCG*) and alpha-fetoprotein (*AFP*).^2^^,^^3^ Isochromosome 12p gain via *in situ* hybridization or polymerase chain reaction is well documented in these tumors and confirms ancestry from a germ cell lineage.^4^^,^^5^ Prior reports have described the emergence of varied histologies with morphology akin to epithelial or endothelial origin carcinomas, mesenchymal origin sarcomas, small round blue tumors subtypes, and rare evolution to even hematologic malignancies.^6–11^

The dedifferentiation of teratoma into a more aggressive phenotype, with increased metastatic potential and insensitivity to both chemotherapy and radiation, underlies the rationale for complete surgical resection.^12^ SM represents a major therapeutic challenge in an otherwise highly curable disease due to the lack of serum tumor markers and limited understanding of its molecular drivers.^13–15^ Our understanding of available therapeutic options is constrained by its infrequency, coupled with most studies reflecting patients treated in the 1980s.^7^^,^^12^^,^^16–19^ Aside from limited evidence supporting anthracycline-based CAV/IE regimens for embryonic-type neuroectodermal tumors (ENET), no established systemic therapy guidelines exist; patients receive either histology-directed or conventional GCT therapy without clear guidelines.^7^^,^^12^ Metastatic disease at presentation, late relapse, higher tumor grade, and residual SM following surgical consolidation have been associated with poor outcomes.^6^^,^^7^^,^^17^^,^^18^ Complete surgical resection has been associated with improved outcomes, remaining the cornerstone of management but is prone to selection bias.^7^^,^^18^

The site- and histology-specific patterns of SM, as well as the time of onset, are not well documented.^7^^,^^8^^,^^13^^,^^16^ Furthermore, its molecular landscape remains unexplored. Here, we characterize the clinicopathologic and genomic features of SM, focusing on site- and time-specific patterns, outcomes, and molecular aberrations.

## Methods

A retrospective review of our institutional database of GCTs including all patients with SM from June 2016 to May 2025 was conducted (Figure S1, see online supplementary material for a color version of this figure). Inclusion criteria required either:

1. Concurrent presence of SM and GCT elements (teratoma, yolk sac tumor, embryonal carcinoma, choriocarcinoma, seminoma) in the same specimen, or
2. Positivity of isochromosome 12p fusion in the current tumor specimen, history of GCT in the past and strong clinical-pathological concordance.

All histologic specimens were reviewed by dedicated genitourinary and thoracic pathologists. All available next-generation sequencing data were abstracted for analysis. Primary platforms included the MD Anderson Molecular Actionable Profiling Panel (MDA MAPP) and the BostonGene Tumor Portrait platforms. The MDA MAPP is an in-house panel with variant detection, which gradually evolved in extent and depth, from a 51 gene panel to its current form, spanning more than 700 genes.^20^ The BostonGene Tumor Portrait test (BostonGene Corporation, Waltham, Massachusetts) analyzes over 20 000 genes, with assessment of microsatellite instability status, tumor mutational burden, germline and somatic variants, tumor microenvironment, and RNA expression.^21^ Genomic data from other platforms and circulating tumor DNA (ctDNA) testing using the Signatera assay (Natera, Inc. Austin, Texas), were included, when available.^22^

Th*e* time to somatic transformation (TST) was analyzed since the initial GCT diagnosis. SM was classified based on the time of first detection: (1) *de novo* if detected at initial diagnosis (TST = 0), (2) detected at consolidative resection after initial chemotherapy, (3) identified at relapse within 5 years, and (4) evolved SM, identified after 5 years from original GCT diagnosis consistent with the definition of very late recurrence of GCT.^23^ For *de novo* SM, “therapy resistance” was defined if progression occurred within 3 months of completion of initial systemic therapy (*progression-free interval [PFI]* *<* *3 months*), consistent with the International Prognostic Factor Study Group (IPFSG) criteria.^24^

Descriptive statistics summarized categorical and continuous variables. The chi-square test or Fisher exact test and logistic regression evaluated associations between categorical variables.

Survival outcomes were analyzed using Kaplan-Meier estimates, log-rank tests, and Cox proportional hazards regression. The impact of ENET histology was assessed from the time of first detection of SM to last follow-up or death. For relapsed SM, the prognostic relevance of therapy resistance and salvage surgery were explored in the *de novo* subgroup, from the time of relapse to last-follow-up or death. Salvage surgery was treated as a time-varying covariate, given its post-baseline nature. The earliest instance of salvage surgery was considered in the case of multiple resections to minimize immortal-time bias.

Data collection was conducted using REDCap, and statistical analyses were performed using R version 4.4.2.^25^ The study was approved by the MD Anderson Institutional Review Board (Protocol no. PA16-0736).

## Results

### Baseline characteristics, histologies, and treatment details

Seventy-two patients were identified, with a median age of 28 years, and 24% were of Hispanic ethnicity. Testicular and mediastinal primaries were observed in 40 (56%) and 24 (33%) of cases, respectively. Forty-two patients (58%) presented with *de novo* SM, while 12 patients (16.6%) had *evolved* SM (detailed breakdown in Table 1) Among those with *de novo* SM, 28 (66%) had metastatic disease at diagnosis, and 18 (42%) had poor-risk disease as per International Germ Cell Cancer Collaborative Group (IGCCCG) criteria,^26^ largely due to the enrichment of mediastinal non-seminomas in this cohort. Of the 11 patients with SM detected at consolidation, 8 had mediastinal primaries, 2 patients had evidence of SM at delayed orchiectomy, and 1 had mature teratoma in the orchiectomy specimen with ENET detected at RPLND.

**Table 1:** Baseline characteristics.

Variable		*N* (%)
**Age at SM diagnosis in years (median; IQR)**		30.8 (24-40)
**Race**	White	54 (75)
	Black	4 (6)
	Asian	3 (4)
	Other	11 (15)
**Ethnicity**	Hispanic/Latino	17 (24)
	Not Hispanic/Latino	52 (72)
	Unknown	3 (4)
**Primary site**	Testis	40 (56)
	Retroperitoneum	2 (3)
	Mediastinum	24 (33)
	Thyroid	2 (3)
	Prostate	2 (3)
	Paraspinal	1 (1)
	Ischiorectal fossa	1 (1)
**Specimen at initial diagnosis of SM**	Biopsy	28 (39)
	Surgical specimen	44 (61)
**Stage for testicular GCT at initial diagnosis**	1	10 (25)
	2	8 (20)
	3	21 (53)
	Incomplete records	1(3)
**IGCCCG risk at SM diagnosis (*n* = 60)** ^a^	Good	21 (35)
	Intermediate	1 (16.6)
	Poor	38 (63.3)
**Time of detection of SM**	*De novo*	42 (58.3)
	Consolidation	11 (15.2)
	Relapse (within 5 years)	7 (9.7)
	Evolved (more than 5 years after initial diagnosis)	12 (16.6)

a IGCCCG risk assigned only for metastatic GCT. Six patients had Stage 1 disease at SM diagnosis. Staging not available for thyroid, prostatic, paraspinal, and ischiorectal fossa primaries (6 patients).

**Figure 1.**
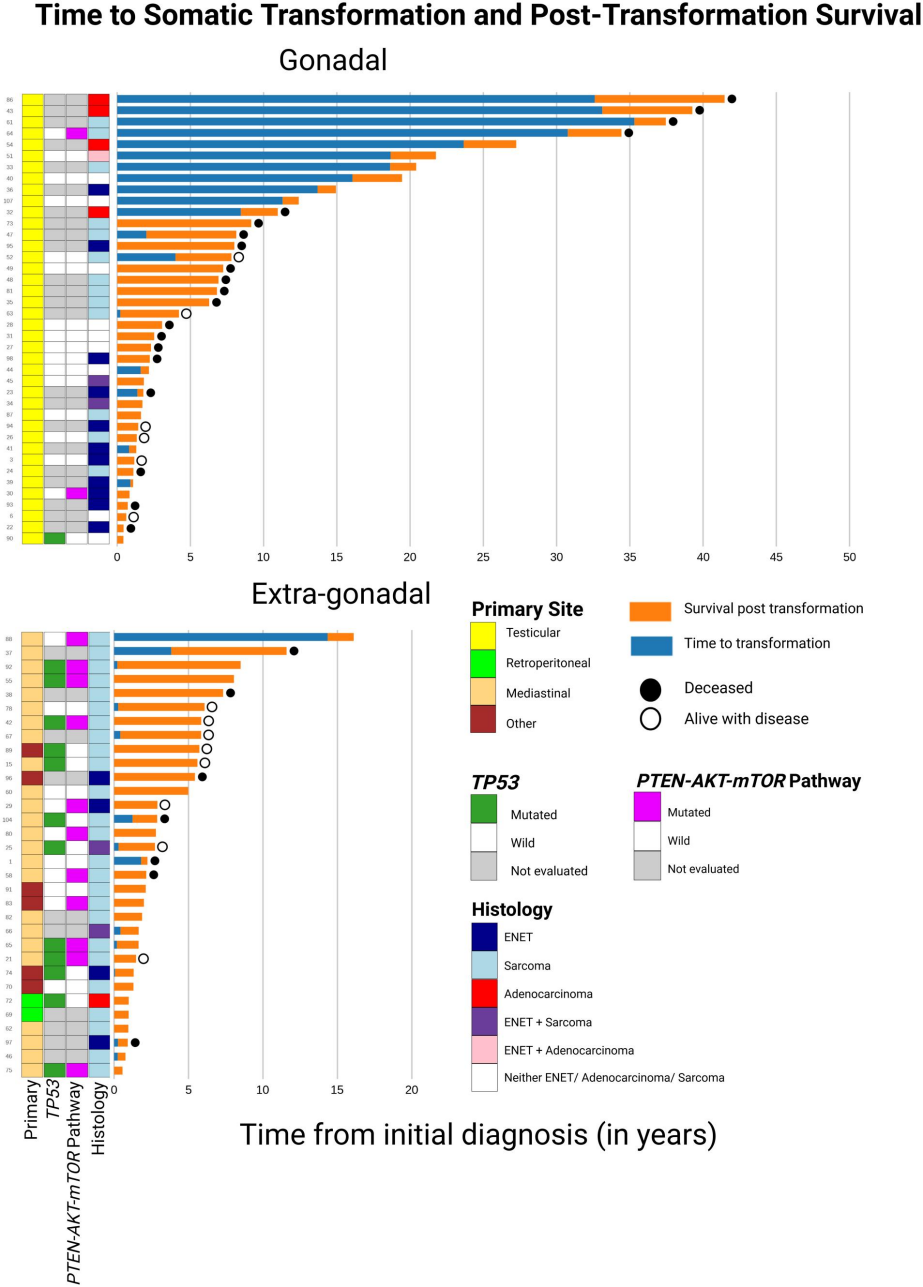
Time to somatic transformation and post-transformation survival according to primary tumor site, histology, and molecular alterations. Swimmer plots depicting the clinical course of patients with somatic-type malignancy (SM) arising from germ cell tumors, stratified by gonadal (left) and extra-gonadal (right) primary sites. Each horizontal bar represents an individual patient. Each bar consists of two sequential segments representing the interval from initial germ cell tumor diagnosis to detection of somatic transformation, followed by survival after somatic transformation. Patients are ordered by total follow-up duration. Annotated columns display primary tumor site, dominant histology, *TP53* mutation status, and alterations in the *PI3K–AKT–mTOR* pathway. Filled circles indicate death, whereas open circles indicate patients alive at last follow-up. Left (gonadal): testicular SM demonstrates a relatively bland mutational profile. Adenocarcinoma histology is predominantly observed in late-evolving tumors with prolonged latency from initial diagnosis, whereas ENET tends to occur earlier and follows a more aggressive clinical course. Right (extra-gonadal): extra-gonadal germ cell tumors frequently demonstrate *de novo* sarcomatous dedifferentiation, characterized by enrichment of *TP53* alterations. Survival following somatic transformation appears more limited in this subset compared with their gonadal counterparts. Abbreviations: SM, somatic-type malignancy; ENET, embryonic-type neuroectodermal tumor; PI3K, phosphoinositide 3-kinase; AKT, protein kinase B; mTOR, mechanistic target of rapamycin.

Sarcomatous transformation was the most common entity (42, 58%) detected followed by ENET (20, 28%). For 10 (14%) patients, at least 2 distinct SM entities were identified at some point during treatment (Figure S2, see online supplementary material for a color version of this figure). The majority of SM detected in patients with mediastinal primaries contained sarcomatous components (22/24, 92%). Among all patients with *evolved* SM, the median TST was 18.6 years [interquartile range (IQR), 14.1-31.2]. Rhabdomyosarcoma (RMS) and ENET had an earlier TST, while adenocarcinoma was almost always detected as an *evolved* SM with a delayed TST (Table 2; See Figure 1—exploring timing of transformation, divergent histologies and distinct mutational profiles by site). Phase-wise breakdown of histologies is available in Table S1.

**Table 2:** Primary site, histology, and temporal characteristics.

Site, histology, and evolution				
		Gonadal (*n* = 40) (%)		Extragonadal (*n* = 32) (%)
***De novo* SM**		22 (55)		20 (62.5)
**Consolidation**		3 (7.5)		8 (25)
**Relapse <5 years**		4 (10)		3 (9.4)
**Evolved SM**		11 (27.5)		1 (3.1)
**Transformed Histology^a^**	Any ENET	14 (35)		6 (19)
	Any carcinoma	10 (25)		4 (13)
	Any sarcoma	15 (38)		27 (84)
**Histology with median time to somatic transformation in years (IQR)**				
**Rhabdomyosarcoma**		0 (0-0.06)		
**ENET**		0 (0-0.52)		
**Adenocarcinoma**		21.2 (11-30.37)		
**Overall survival differences in years**				
**Variable**	Median (95% CI)		HR (95% CI)	*P* value
**ENET**	1.8 (1.20-NA)		1.94 (0.87-4.31)	.1
**Non-ENET**	8.0 (3.34-NA)			
**Testicular ENET**	1.8 (1.3-NA)		3.81 (1.28-11.32)	.01
**Testicular non-ENET**	NA (3.6-NA)			
***De novo* therapy resistant**	1.5 (1.1-NA)		2.12 (0.58-7.72)	.2
***De novo* therapy sensitive**	NA (0.9-NA)			
**Salvage surgery**	NA (0.5-NA)		0.64 (0.18-2.25)	.4
**No salvage surgery**	0.9 (0.4-NA)			

a Transformed histologies were recorded as “any ENET” or “any sarcoma” or “any carcinoma.” Because these categories are not mutually exclusive, cumulative percentage may exceed 100%.

### Treatment of *de novo* and evolved SM

Among patients with *de novo* SM, 35/42 (83%) received initial chemotherapy. Platinum and anthracycline-containing regimens were received by 20/42 (47%) and 7/42 (16%) patients respectively, and 5/42 (13%) received both agents. Histology-guided therapies for those with *de novo* SM are listed in Table S2. Complete response (CR) and marker negative partial response (PRm-) for those with *de novo* SM were observed in 5/42 (11.9%) and 12/42 (28.5%) of patients, respectively, while 10/42 (24%) had progressive disease after initial chemotherapy. Initial surgical consolidation was performed for 23/42 (54.7%) patients.

Of the 30 patients who had disease relapse after first-line treatment for *de novo* SM, 21/30 (70%) of patients received chemotherapy and 15/30 (50%) underwent surgery. Salvage radiation was a consolidative strategy for 13/30 (43%) patients.

Among patients with evolved SM (*n* = 12), the retroperitoneum was the most common site of first detection (6/12, 50%). Detailed breakdown of all sites of relapse and response data is in Table S3. Eight of 12 patients had a single site of disease relapse and 7 of them underwent upfront salvage resection.

### Overall survival post transformation

**Figure 2.**
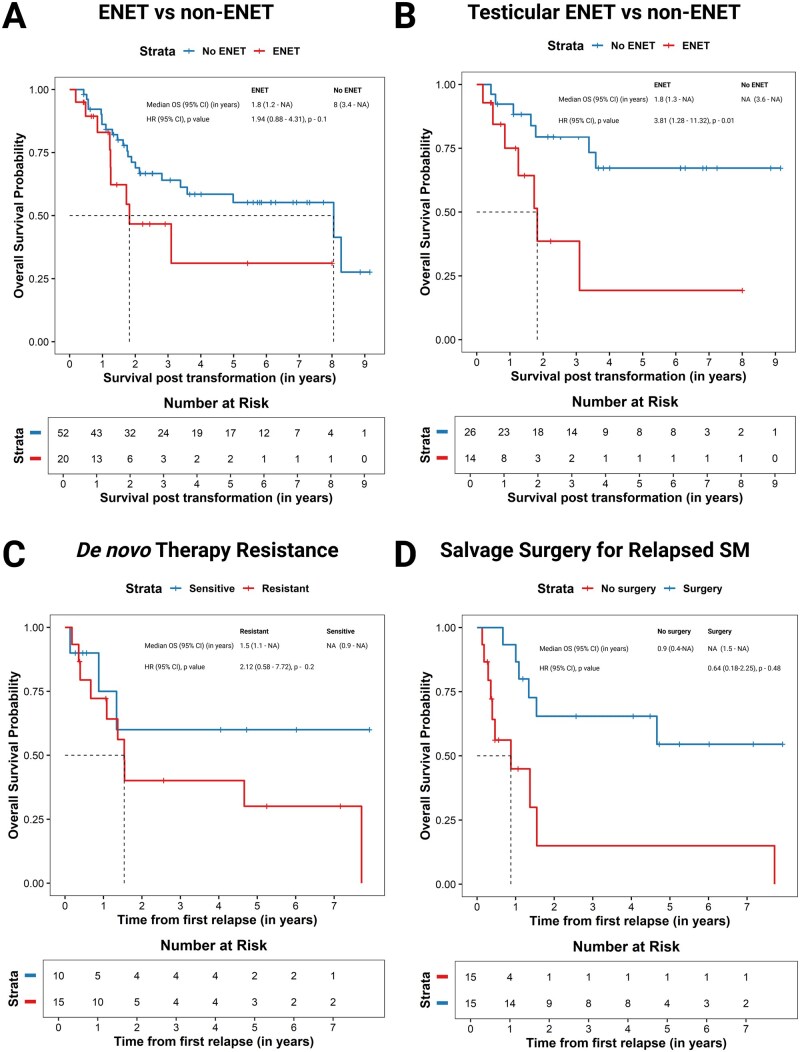
Survival outcomes according to histology, therapy resistance, and salvage surgery in patients with somatic-type malignancy (SM) arising from germ cell tumors. (A) Kaplan-Meier estimates of overall survival (OS) from the time of somatic transformation comparing patients with ENET-containing SM vs non-ENET histologies. (B) OS from the time of somatic transformation among patients with testicular primary tumors stratified by ENET vs non-ENET histology. (C) OS from first relapse among patients with *de novo* SM according to therapy resistance status, defined as relapse within 3 months of completing frontline systemic therapy. (D) OS from first relapse among patients with relapsed SM according to receipt of salvage surgery, modeled as a time-dependent exposure. Median OS, hazard ratios (HRs), 95% confidence intervals (CIs), and *P* values are displayed within each panel. Tick marks indicate censored observations. Dashed lines denote median survival estimates. Number-at-risk tables are shown below each curve. Abbreviations: ENET, embryonic-type neuroectodermal tumor; SM, somatic-type malignancy; OS, overall survival; HR, hazard ratio; CI, confidence interval.

At the time of analysis, 38% of all patients had no clinical evidence of disease, 19% were alive with disease, and 43% were deceased. ENET histology was associated with a shorter OS compared to non-ENET cases (median OS, 1.8 years vs 8 years; *P* = .1). Notably, the survival of testicular ENETs was significantly shorter compared to testicular non-ENET histology (median OS, 1.8 years vs not reached; *P* = 0.01) (Table 2 and Figure 2).

After relapse, therapy resistance among patients with *de novo* SM correlated with shorter OS (median OS, 1.5 years vs not reached; *P* = .2) while salvage surgery associated with longer OS (median OS, not reached vs 0.9 years; *P* = .4); however, these analyses are exploratory and sample size is small (Table 2 and Figure 2).

### Molecular correlatives

Somatic genomic data was available for 39 patients, taken from 23 primaries and 16 metastatic specimens (see Supplementary Sheet). Primary specimens with *TP53* mutations detected at any point were observed in 13/39 cases (33%), almost exclusively in extragonadal primaries and sarcomatous transformations. *PI3K-AKT-mTOR* pathway alterations involving *PTEN, AKT, PIK3CA, PIK3CD, PIK3R1, mTOR, TSC1/2*,^27–29^ were observed in 13/39 (33%) cases, with similar predominance (>85%) in primary extragonadal disease. These mutations were associated with sarcomatous transformation compared to non-sarcoma though confidence intervals are wide (OR, 5.27; 95% CI, 0.88-58.2; *P* = .04) (Figure 1). KRAS/NRAS mutations were seen in 7/39 (18%).

### Circulating tumor DNA

Circulating tumor DNA (ctDNA) was assessed in 3 patients, all of whom had normal serum tumor markers. At relapse, ctDNA was detectable in all of them prior to salvage therapy. In 2 patients with mediastinal sarcomatous SM, ctDNA became undetectable following treatment and remained negative during follow-up; 1 patient awaits salvage therapy initiation. Conventional tumor markers remained negative throughout surveillance in these patients.

## Discussion

This study highlights fundamental distinctions among SM histologies according to the timing of onset, primary site, and mutational status. The observed outcomes reflect contemporary treatment patterns at a tertiary referral center in the United States.

Of particular importance are the temporal patterns of SM detection. While categorization was performed using 4 time points, these broadly fall into 2 distinct disease biologies: earlier transformation (*de novo*, consolidation, or early relapse) enriched for sarcoma, and later transformation (evolved SM), in which adenocarcinoma is detected. This hypothesis is supported by the histologic patterns across these groups (see Table S1).

Notably, sarcomatous SM, particularly rhabdomyosarcoma (RMS), was enriched within these early-presentation groups, further supporting its association with mediastinal primaries, which are frequently detected upfront.^7^^,^^16^ Previous studies have similarly demonstrated a predominance of RMS among sarcomatous SM, with more than half arising from extragonadal primaries.^14^^,^^30^ In the largest series on SM to date, 5% of transformations originated within mediastinal primaries, but further histological characterization was not provided.^7^

In contrast, evolved SM may represent a biologically distinct entity. Rice et al reported that 37% of patients with SM underwent reoperative RPLND, underscoring the strong association between evolved SM and previously treated disease.^16^ Consistent with this observation, more than 65% of patients with evolved SM in our cohort had received prior GCT-directed therapy between 1980 and 2005 and subsequently developed transformation after a median TST of 18 years. These findings support the hypothesis that evolved SM may arise as the eventual consequence of longstanding unresected residual teratoma. Accordingly, it is unsurprising that evolved SM constitutes only a minority of this contemporary cohort, with notable sparsity among extragonadal primaries (Figure 1), for which aggressive post-chemotherapy surgical consolidation is the standard of care.^31^

Our findings also demonstrate a strong association between adenocarcinoma histology and evolved SM. Prior reports have similarly observed adenocarcinomatous transformation predominantly at metastatic sites and in late recurrences occurring 15-20 years after the initial GCT diagnosis.^7^^,^^32^

Among non-ENET histologies, response rates to initial chemotherapy have been reported at 13%.^16^ Our CR rate of 11.9% also suggests that these tumors are inherently chemotherapy resistant. In comparison, conventional GCT demonstrates CR rates as high as 70%.^33^ Notably, our study included ENETs, an entity with limited response to systemic therapy.^12^ In contrast to the favorable outcomes and success seen in conventional testicular GCT, the particularly poor response and OS observed with testicular ENET underscores the adverse prognostic implications of this histology, irrespective of the site of origin.

In our cohort, a PFI of less than 3 months following initial chemotherapy correlated with shorter survival in the relapsed setting among patients with *de novo* SM, mirroring the outcomes with conventional GCT, but the small sample size limits our conclusions.^24^ Similarly, and without adjustment, salvage surgery correlated with longer survival. However, the early separation of curves likely reflects favorable clinical factors such as performance status, localized disease, sites of metastases, and resectability, that contribute to this association. While the time-dependent modeling of salvage surgery adjusts for guarantee-time bias, upfront selection likely persists.

Our cohort was also noted to be enriched for *TP53* mutations in the mediastinal SM subgroup. Mutated *TP53* is uncharacteristic of testicular GCT, but is distinctly observed among mediastinal primaries, and have been linked to cisplatin resistance.^14^^,^^34–36^ Given the degree of overlap between sarcomatous SM and mediastinal origins in our dataset, these mutations likely represent the site of origin rather than the phenomenon of somatic transformation, which remains poorly understood. *PTEN-AKT-mTOR* pathway mutations included alterations of the following proteins: *PTEN, AKT, mTOR, TSC1/2,* and *PI3K* complex.^27–29^  *PTEN* is frequently altered in GCT, marking the transition from neoplasia *in situ* to invasive disease.^14^^,^^37^ Notwithstanding the limited numbers, these mutations were also consistently observed in mediastinal sarcomatous SM, and are particularly noteworthy given the potential for targeted therapies in relapsed disease.

Recently, ctDNA was shown to be a sensitive and reliable monitoring tool in nonteratoma GCT. Unlike tumor markers, the presence of ctDNA during surveillance window additionally conferred a shorter time to disease progression.^38^ The literature on the utility of ctDNA in marker-negative GCT, particularly teratoma and SM, is limited.^39^ A recent study of 92 patients with retroperitoneal disease, reported impressive correlation between ctDNA status and active GCT on histology, but a dramatic drop in predictive value for patients with pure teratoma detected at surgical resections.^40^ It is unclear whether patients in this study harbored any SM component. In our series, 3 of 3 patients with SM had detectable ctDNA, and 2 cleared ctDNA with salvage resection while one awaits treatment. These results are preliminary and warrant larger prospective investigation.

As with prior studies of SM, the phase-wise delineation of histologies in this report was appreciated retrospectively. However, there are currently no available biomarkers to predict transformation of teratoma to SM. This is an unexplored area that may guide post-chemotherapeutic management of residual masses. Future efforts must be directed toward distinguishing post-chemotherapeutic residual teratoma with SM causing early relapse from those at risk of late evolution, versus those that may never relapse.

Additionally, the heterogeneity and limited size of the cohort constrain our capacity to discern differences among the various management strategies. Further analysis of regimen activity (platinum vs anthracycline-based therapy) in sarcomatous SM was not feasible. Only unadjusted survival analyses were performed due to the modest sample size. Mutational data presented in this report were available for only a subset of patients and the findings are descriptive. We acknowledge that sequencing panel heterogeneity and depth of screening can affect interpretation of mutational data. We also concede the potential referral bias that may affect the mixture of SM histologies.

To our knowledge, this single-center study is among the first to delineate important site-based and temporal distinctions in SM. There is considerable representation of extragonadal primaries, which have not been extensively reported in prior series. The adverse outcomes observed with ENET histology compared to non-ENET variants attest to the reproducibility of results of prior reports, and the reliability of the current body of evidence regarding SM.

## Conclusion

In summary, SM exhibits distinct temporal, anatomic, and molecular patterns. These findings suggest that SM represents a biologically diverse entity with site- and histology-specific characteristics. External validation is warranted, and larger studies are needed to define optimal systemic treatment strategies.

## Supplementary Material

oyag253_Supplementary_Data

## Data Availability

Data will be available upon request.
