# Supplementary material for: Divergent biology and outcomes of somatic transformations in germ cell tumors
Source: Oncologist. 2026 Jun 30;31(8):oyag253. doi: 10.1093/oncolo/oyag253 (PMC13364673; doi:10.1093/oncolo/oyag253)
Supplement: oyag253_Supplementary_Data [file oyag253_supplementary_data.zip › Supplementary Table 3.docx]

**Supplementary Table 3: Evolved SM – Sites of relapse at detection of SM, Response to systemic therapy**

| **Evolved SM *(n=12)*** | n (%) |
| --- | --- |
| *Sites of metastases ** |  |
| Retroperitoneum | 6 (50) |
| Posterior Mediastinum | 1 (8.3) |
| Other lymph nodes | 1 (8.3) |
| Lung | 4 (33.3) |
| Bone | 1 (8.3) |
| Pleura | 1 (8.3) |
| Colon | 1 (8.3) |
| Pancreas | 1 (8.3) |
| *Response to treatment* |  |
| PR | 1 (8.3) |
| SD | 1 (8.3) |
| PD | 1 (8.3) |
| Surgical resection | 7 (58.3) |
| Not available | 2 (16.6) |

* - Metastatic sites are not mutually exclusive and so % may exceed 100.
