# Supplementary material for: Divergent biology and outcomes of somatic transformations in germ cell tumors
Source: Oncologist. 2026 Jun 30;31(8):oyag253. doi: 10.1093/oncolo/oyag253 (PMC13364673; doi:10.1093/oncolo/oyag253)
Supplement: oyag253_Supplementary_Data [file oyag253_supplementary_data.zip › Supplementary Table 1.docx]

Supplementary Table 1 – Phase-wise breakdown of SM histologies

| Histology | ENET (n=20) (%) | Sarcoma (n=42) (%) | Adenocarcinoma (n=6) (%) |
| --- | --- | --- | --- |
| *De novo* | 12 (60) | 25 (59.5) | 1 (16.6) |
| Consolidation | 4 (20) | 8 (19) | 0 |
| Relapse under 5 years | 2 (10) | 5 (11.9) | 0 |
| Evolved | 2 (10) | 4 (9.5) | 5 (83.3) |

ENET- Embryonic-type neuroectodermal tumor
