# Supplementary material for: Divergent biology and outcomes of somatic transformations in germ cell tumors
Source: Oncologist. 2026 Jun 30;31(8):oyag253. doi: 10.1093/oncolo/oyag253 (PMC13364673; doi:10.1093/oncolo/oyag253)
Supplement: oyag253_Supplementary_Data [file oyag253_supplementary_data.zip › Supplementary Table 2 clean.docx]

**Supplementary Table 2: Histology- guided systemic regimens used for *de novo* SM**

| Sno. | Histology guided systemic regimens* | Histology |
| --- | --- | --- |
| 1 | Etoposide-Cisplatin x 2 + Capecitabine-Oxaliplatin x 4 | Adenocarcinoma |
| 2 | ATP x 2 + Doxorubicin-Ifosfamide x 2 | Angiosarcoma |
| 3 | Doxorubicin-Ifosfamide x 6 + VIP x 4 |  |
| 4 | Alectinib x 5 weeks | Carcinosarcoma |
| 5 | VAI X 6 | Pleomorphic sarcoma |
| 6 | VAC/IE X 6 | PNET |
| 7 | VAC/IE |  |
| 8 | VIP + VAC X 8 + Pembrolizumab |  |
| 9 | VDC X 6 | RMS |
| 10 | VI/VDC/IE/VAC |  |
| 11 | VAC X 5 |  |
| 12 | Doxorubicin-Ifosfamide x 4 | Spindle cell sarcoma |
| 13 | ADIC x 2+ VIP x 2/EP x 2 | Undifferentiated sarcoma |
| 14 | Doxorubicin -Ifosfamide-Cisplatin x 6 |  |
| 15 | Doxorubicin-Ifosfamide x 10 + Gemcitabine-Docetaxel x 2 |  |
| 16 | Dactinomycin/ Doxorubicin/Vincristine- Cyclophosphamide/Etoposide x 31 weeks | Wilms Tumor |

*- Regimens were individualized and informed by available histologic and molecular data available

Abbreviations: ATP: Doxorubicin-Paclitaxel-Cisplatin; EP: Etoposide-Cisplatin; Xelox: Capecitabine-Oxaliplatin; VIP: Etoposide-Ifosfamide-Cisplatin; VAI: Vincristine-Dactinomycin-Ifosfamide; VAC: Vincristine-Doxorubicin-Cyclophosphamide; IE: Ifosfamide-Etoposide; VDC: Vincristine-Dactinomycin-Cisplatin; VI: Vincristine-Irinotecan; ADIC: Doxorubicin-Dacarbazine-Ifosfamide-Cisplatin
