# Supplementary figures and images for: Divergent biology and outcomes of somatic transformations in germ cell tumors
Source: Oncologist. 2026 Jun 30;31(8):oyag253. doi: 10.1093/oncolo/oyag253 (PMC13364673; doi:10.1093/oncolo/oyag253)

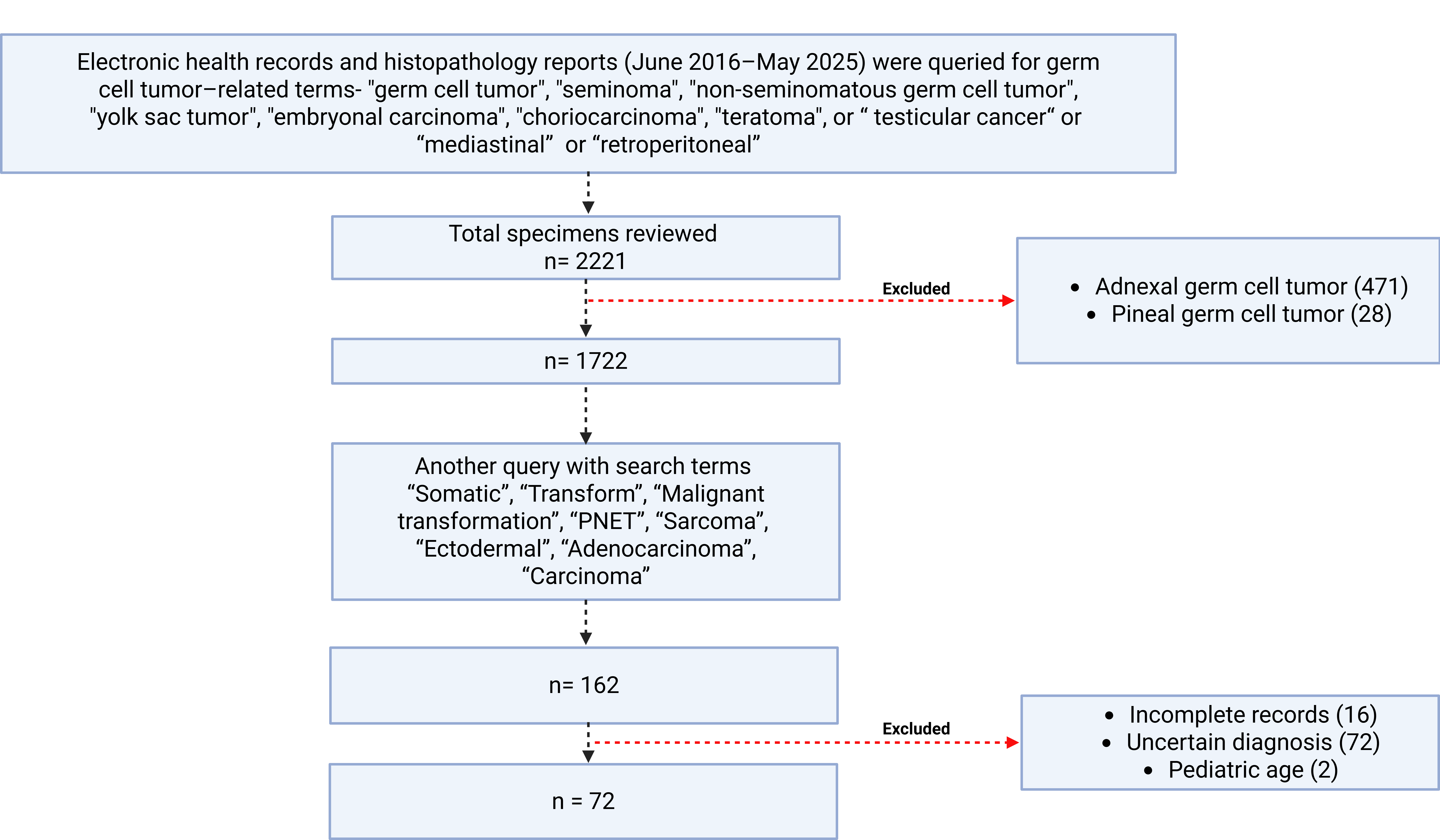

Supplement: oyag253_Supplementary_Data [file oyag253_supplementary_data.zip › Supplementary Figure 1.png]

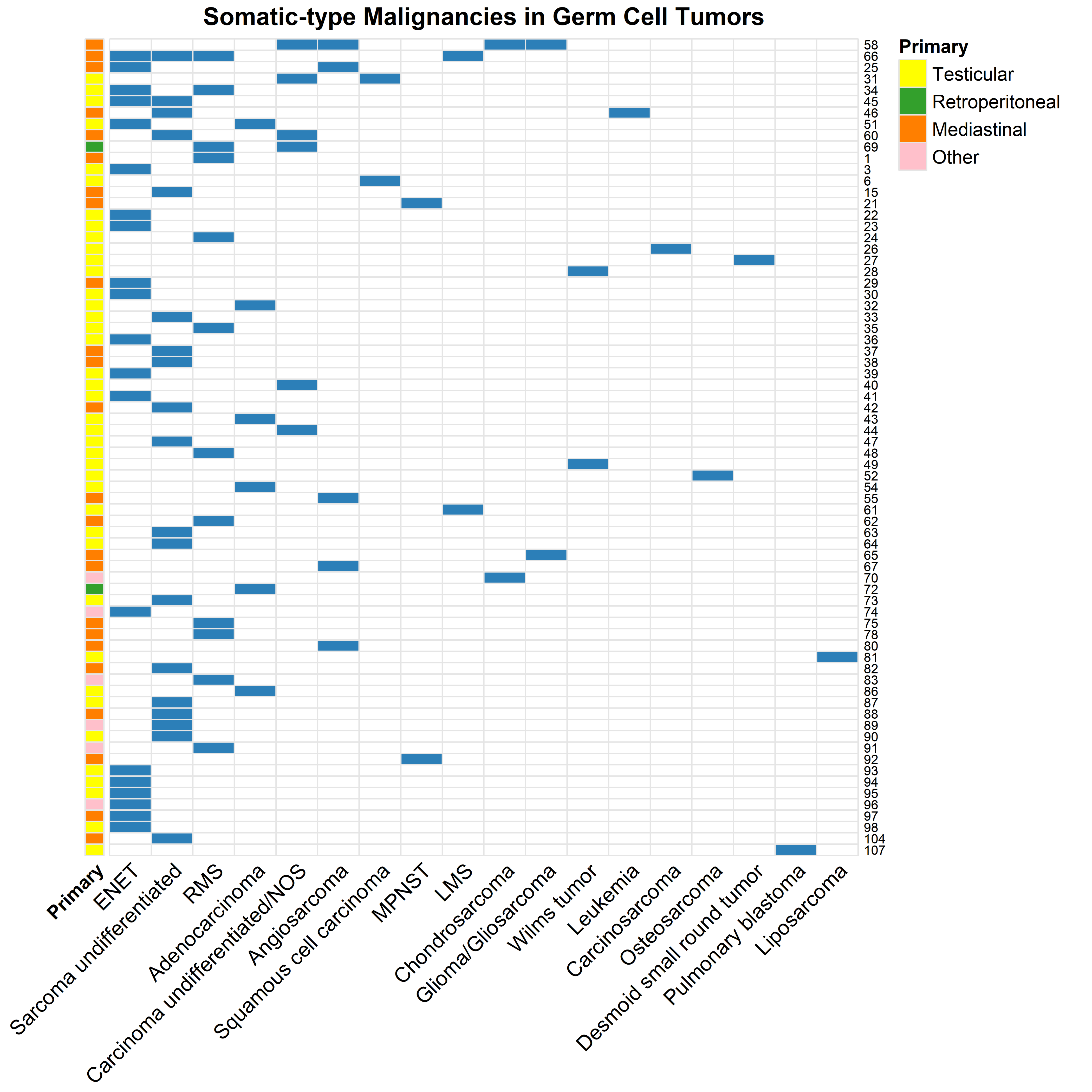

Supplement: oyag253_Supplementary_Data [file oyag253_supplementary_data.zip › Supplementary Figure 2.png]
